# Observations on Solar Asphyxia, Coup De Soleil or Sun Stroke

**Published:** 1841-11

**Authors:** B. Dowler

**Affiliations:** New Orleans


*Observations on Solar Asphyxia, Coup de Soleil or Sun-Stroke.* By B. Dowler, M. D., of New Orleans.—In the numerous works written or re-published in the United States, I have not seen any satisfactory notice of this very frequent, and in the south, most fatal of all maladies. During five weeks, ending with last month, (July,) five fatal cases occurred in my practice alone.

The name I have chosen for the disease, (*Solar Asphxyia,*) being descriptive of its leading symptoms, will, I trust, receive the sanction of the profession and the public. I adopted it several years before I had an opportunity of testing by dissections, the character of the disease, which it so fully expresses. *Pulmonary apoplexy*, is a name, more characteristic of the morbid appearances of the lungs, but it makes no allusion to its solar origin, or to the symptoms of suffocation, which mark its progress.

*Solar diseases* require a particular arrangement or classification which is highly important in a practical point of view. I submit the following:—

I. *Solar exhaustion or syncope;*
II. *Solar or sun-pain;*

III. *Solar excitement or inflammation;*
IV. *Solar asphyxia;*

which we propose to make the principal subject of our investigation.

I. Solar exhaustion differs from solar asphyxia, both in symptoms and treatment. In solar asphyxia, the skin is extremely hot, and generally dry; there is a choking sensation and a total loss of sense.

In solar exhaustion, the skin is moist, pale and cool; the breathing is easy, though hurried; the pulse is small and soft; the vital forces fall into a temporary collapse, the senses remaining entire. Horizontal position, free air in the shade, external stimulants and frictions, are usually sufficient to restore the patient. Vomiting is very useful, and is easily excited, as there is usually nausea; warm water, and if necessary, a few grains of ipecac. may be given. Vomiting appears to throw the blood from the centre to the circumference. The pulse rises.

That fatal disease of northern summers, ascribed to drinking cold water, is probably, nothing but solar exhaustion, in which cold water has but a secondary agency. It happens only in very hot weather.

II. Solar, or as it is commonly called by the people, sun-pain, is a chronic disease in which the abdominal organs are more or less deranged, though the most remarkable symptom is a pain in the head, while the sun is above the horizon.

III. Solar excitement or inflammatory re-action, sometimes follows solar asphyxia of the second degree, being attended with febrile heats, arterial throbbings and headaches.

*Case.*—A cooper while working in the sun, fell so suddenly as to bruise and lame himself considerably. He soon got up; but feeling feverish, and having pulsations in the head, for several days he tried the Thompsonian treatment without benefit. He called on me for advice. He was bled freely, which caused a long continued syncope, from which he arose cured. He took no medicine and went to work the next day.

J. E. had been to the town of Bath, six miles distant, on the 4th day of August, 1837, in company with Dr. R. and another person; while attempting to walk back to New Orleans, upon the rails of the Nashville rail-road, not then finished, the doctor fell sun-struck, and expired in a few minutes. The body was abandoned. The other companion of E. had not walked far, when he too fell, and died in like manner. The next morning as E. was passing opposite my door, on the sunny side of the street, he fell. I instantly bled him, upon the pavement. The blood trickled slowly at first, but soon after flowed in a good stream, and the pulse, though feeble, became fuller during the operation. In a few minutes, the man walked to his boarding-house, without feeling any unpleasant symptoms; but a moderate re-action coming on during the day, cathartics were given, and by the following day, his health was restored. In this case, blood-letting was probably useful, but in nine cases out of ten, it is useless if not worse, accelerating the death from five to fifteen minutes. During the last five years, I have been called to see a very considerable number of sun-struck persons, within five or ten minutes after they fell in the streets: formerly, I used to bleed them, and though the great heat of the body is thereby suddenly diminished, the pulse becoming soft as air, yet by the time the arm is tied up, (which is done more for form than necessity,) the patient is choked suddenly, and to appearance, by a dense tenacious mucus, the breathing not ceasing gradually, as in other disease, but instantly the face turning livid, and even its veins, especially upon the forehead, becoming at the moment distended. Bleeding hastens the strangulation, though it is always desired by the friends.

Without anticipating the description of sun-stroke, to be found in the sequel, I may add another variety, a sub-acute affection, beginning with solar excitement and ending in asphyxia.

*Case.*—A gentleman who was not acclimated, exposed himself to the sun until noon, when he sent for a physician, who bled him most profusely, (two pounds,) gave him cathartics, applied mustard on the stomach and legs, cold to the head, &c. Afterwards he walked about for some time, but in the afternoon became insensible. About sun down I was called in, and found him asphyxiated, unable to swallow, totally insensible, breathing as in sun-stroke; pulse air-like and quick; the skin hot. He did not die until late in the night. This affection lasted about fifteen hours; more than fifteen times longer than the average duration of acute solar asphyxia, which has but one stage, as truly as hanging, drowning or suffocation from carbonic acid gas, with this difference, that in the former there is not a movement in any of the muscles, except those concerned in breathing and in circulating the blood, except immediately before or after the last breath, when there is sometimes, a very slight contraction of the fingers, and a kind of bending or turning of the body towards one side, though this is scarcely observable.

Solar exhaustion in its mildest and chronic form, that of debility, is a uniform effect of our hot season, until after acclimation. During this period, though the health may be good, the ability to perform the usual amount of labor is diminished. Even horses are debilitated, often get the *thumps*, and frequently die from solar asphyxia, during the acclimating period. This is so well known that an acclimated horse or mule is worth much more than one not so protected.

Slaves from Missouri, Virginia and Maryland, suffer as much if not more from debility, than the whites, during the first and second summers. I have seen some whose tongues were pale and flabby, whose pulses were feeble and irregular. In these cases the muscular power is lessened; the skin is covered with an abundant, cool perspiration, and sometimes, there are palpitations of the heart, not unlike those which attend organic, rather than functional disorders of that organ, requiring the horizontal position, than which nothing is more important for the removal of these affections during solar acclimation.

Solar and terrestial heat, differ essentially in their action; on health the latter seems never to produce any morbid effects, resembling sun-stroke, though many firemen in boats, foundries and furnaces, are exposed to a high temperature. The solar rays may undergo some unknown modifications from local causes, independent of mere calorific influence.

In Louisiana, solar asphyxia is rather an *urban* than a *country* disease, affecting those not thoroughly acclimated to the city. The persons alluded to in the following extract, were doubtless unacclimated. "In 1821, H. M. Frigate Liverpool, was proceeding from Muscat to Bushire, the weather gradually increased in warmth, double awnings were spread, the decks kept constantly wetted, and every precaution used to prevent the exposure of her men; yet in one day, from a *species of coup de soleil,* she lost three lieutenants and thirty men. If, for however brief a period, they exposed themselves to the sun, they were struck down senseless. The frigate's main deck at one time, is described to have resembled a slaughter house, so numerous were the bleeding patients. (Travels by J. R. Wellstead, Esq., F. R. S. F. R. A. S. vol. i. p. 75, 1841.)

Upon the 24th of May, 1839, after visiting a man who fell asphyxiated, in the St. Mary's Market, and who lived only twenty minutes, I called at another place to visit a patient, and while in the house, a man of very robust appearance, fell with great force upon the floor, causing the blood to flow from his nostrils freely. He soon recovered, and informed me that he had been exposed to the sun, and had fallen once before during the day, but feeling very well as he said, he declined the offer of my medical services. I met him daily for some time after this occurrence, in good health. He declared that no pain or inconvenience attended his falls except some bruises.

Solar asphyxia, sometimes has taken place in the shade, immediately after exposure to the sun, and, even as late as five or six o'clock in the evening. In several instances, the patients came in at five o'clock, P. M., and fell unknown to any one, and were not discovered in their rooms for an hour or two. Attacks late in the day are not so quickly fatal, as those occurring between noon and the middle of the afternoon.

If the attack happen in the hottest part of the day, it terminates in death, in about half an hour. July 26th, 1838, at 3 o'clock, P. M., when the thermometer placed against a brick wall, upon which the rays of the sun struck obliquely, stood at 130°, I was called to visit a paver, a stout middle aged man, who fell in Fourcher street, near my office. I saw him within five minutes after his fall, his skin intensely hot, breathing noisy, irregular and with subdued sobbings, unable to swallow, pupils rather contracted, eyelids nearly closed, pulse extremely variable, irregular and quick. A vein being opened, the blood jetted and stopped alternately, the pulse becoming gaseous as the blood flowed. Ice water was poured upon the head and neck, a mustard paste was spread over the body. He lived thirty minutes, expiring upon the pavement.

If the attack should happen late in the afternoon, it may last from one to two hours, or even longer. I have had at least five to six cases, in as many years, where persons have not long before sunset quit work on account of the heat, rather than from any sickness, they have returned to their rooms, when an hour or more after, they have been found accidentally, in a dying state from solar asphyxia, not having made any noise to arrest the attention of the family.

The most usual place of attack, is among paved streets and brick walls, or upon the levee, where there is no shade. The structure of our wharves and levees, would admit of many shade trees, without interfering with the utilitarian cravings of commerce. Such an improvement would be alike ornamental and useful.

Almost the only persons subject to this malady, are white males, who labor in the sun, and who have not passed through the acclimating period of three or four years. I never saw but one negro die from this cause. He fell in Julia street, Aug.

2d, 1837, and lived about an hour. He was very stout, but I do not know whether he had been acclimated. A negress, while at the washtub, was struck down. I found her senseless, speechless, and breathing with some difficulty, but she retained the power of swallowing; and by the use of cathartics, sinapisms and blisters, recovered in two days.

It is often impossible to get exact histories of the premonitory symptoms. In some instances the patient has not probably had any. The history of the case is something like the following. He had eaten his dinner as usual at noon, urged by his wants or love of gain, he went forth to brave the heat of the sun. The walls, roofs and pavements, now heated to the utmost radiate an intense heat, the temperature exceeds that of the human body, from 30° to 40°. The laborer, perhaps, wears a thin straw hat, and has his hair cut close, in order to keep the head cool; this in fact, exposes the head to the solar influence much more than a thick coat of hair, and a wool hat would do; from the same false theory, he wears a thin cotton shirt, which is the only garment with which his chest is covered, and which, when saturated with sweat, affords but a feeble resistance to the conduction of heat into his body. Now placed in an atmosphere 30° or 40° hotter than his body, it is plain that a blanket coat, or still better, two shirts, one of good thick flannel or wool, next the skin, and one of cotton over the other, would be a great defence against the sun, affording the coolest kind of dress, except to such persons as are in the shade.

Thus thinly clad, the laborer engages at the hard exercise of rolling cotton bales, loading or of unloading ships, coopering, digging or paving. The exercise increases the influence of the atmospheric heat; he finds his skin becoming hot and dry, the next instant he falls, or perhaps, he may conclude to "knock off" from work. He proceeds homeward, a square or two, before he drops to rise no more; the passers-by collect around him; some run for a doctor, some apply ice to his head, and the first bleeder that can be had, perfoms blood-letting. From the total loss of sensibility in the patient, the first impressions of the physician, lead him into the belief that the malady is apoplexy. Of this, more hereafter.

The patient's mouth is found rather open; the under jaw has fallen; a tenaceous mucus appears between the lips and in the nostrils; the breathing is irregular, unequal, laborious; the chest not expanding and oontracting well, reminds the beholder of the voluntary efforts which patients sometimes make in fractures of the ribs, and pleuretic or rhumatic inflammations, to prevent the movements of the chest, necessary to full breathing. The wind-pipe moves violently up and down, the abdomen and diaphragm rising and falling simultaneously. The breathing is noisy, but not stertorous. The sound may be heard several yards from the patient. Just below the clavicles, the sounds as heard through the stethescope, are very remarkable. Some are acute, some are dull, with a puffing or roaring sound. By applying the hand to the chest, a bubbling or boiling could be felt beneath. As death approaches, these noises recede from the extreme branches of the wind-pipe, and occupy the upper proportions of that tube. At length the accumulating mucus obstructs the passage, an involuntary effort is made to breathe, but in vain; yet sometimes, as in the fatal moment of croup, one or two additional respiratory movements take place. Almost always, the breathing stops *suddenly*, by a strangling fit. Often at the instant of death, or at the instant after the cessation of respiration, the face turns almost black, and the veins of the forehead swell, as if a violent effort was being made to get another breath. Before breathing ceases, retching sometimes take place; or a kind of strangling cough, or an inarticulate moaning like that from some of the dumb animals, when in pain; or perhaps there is a kind of suppressed sobbing or sighing like that caused by sudden immersion in cold water. Yet in everything except respiration, death takes place with the utmost tranquillity, not being accompanied with spasmodic or convulsive distortions, so common in fevers and affections of the brain. Be it what it may, the cause of death begins, continues, and ends in the breathing aparatus.

The pulse is hurried, hobbling and unequal; very often gaseous or air-like, but never slow, hard and large, as in apoplexy and some other diseases. The external veins are not full, and the arteries of the arm are easily compressed by a ligature. When a vein is opened, the blood sometimes trickles, then starts in jets, stopping and starting several times. As the blood flows, the pulse becomes more and more gaseous, the heart diminishes, perspiration begins, and strangulation almost immediately follows.

From the commencement of the disease, the power of swallowing is totally gone in almost every case. Whatever is poured into the mouth, runs out or rattles in the throat, according to the position of the body. I have given a mixture of mustard and salt, or ipecac.; but I am now satisfied, that to put any thing into the mouth, is not only useless, but positively hurtful, as it often drops into the windpipe. The eyes.

are not projecting, discolored or rolling, but generally maintain their parallelism, though they are somtimes turned upward; they are less closed than in sleep; the pupils are perfectly natural; the power of winking is totally lost; the eye is lustreless, and expressive of a dying state. The patient is usually found lying on his back; he has no power to change his position; his neck, body, and limbs are free from rigidity or motion throughout the attack. In the act of dying, I have noticed a slight curving of the body laterally, with a feeble contraction of the fingers. In solar asphyxia, the symptoms, and the manner of death, are more uniform than in any other malady. The heat of the body, both before and after death, is a most remarkable circumstance. In the hurry, incidental to a death so sudden, I have not had an opportunity of applying the thermometer, to ascertain the exact temperature; but judging from the sense of touch alone, it would seem much greater than in the hottest fevers. The heat may be felt, radiating from the patient's body, at the distance of two or three feet. In cases where the patient has not been bled copiously, the heat is very pungent. The heat of the body continues, generally, many hours after death, including the whole night. This is the more remarkable, as our nights are not hot and sultry, but accompanied with breezes, which, by morning, cool even the walls and pavements.

After the death of the lungs, or the cessation of respiration, the heart and arteries will, in some instances, continue to act.

*Case.*—Mr. C. died of solar asphyxia, on the evening of July 24, 1836. About an hour after he had been laid out, two messengers called on me to visit the corpse, which was supposed to be alive. I found the body as warm as at death, though it had since been washed. I found a slight pulsation at the wrist, and a feeble motion of the heart. Dr. Young, of this city, was lately called, under similar circumstances, about two hours after breathing had ceased, from sun stroke. He found the body very hot, and, as he thought, slight motions of the heart and arteries. I dissected two bodies at the same time, that had died the day before, and found the heat of the trunks about equal to that of a person in health.

After death, the face and neck become of a blackish or purplish, spotted hue; a mucus foam, often mixed with blood, begins, soon after death, to issue from the mouth and nostrils, and is very copious, in many cases, the day following.

*So Asphyxia, or Coup de Soleil,* has long been regarded as apoplexy: the entire loss of the senses—the universal paralysis—all seem at first view, to sustain that common, but erroneous, opinion. In the worst cases of apoplexy, the patient is not always instantaneously deprived of volition, feeling, and motion. The apoplectic, in the early stages of the malady, may be awakened, and can answer, though not able to speak more than a word or two. He will generally open his eyes on being spoken to; can swallow, and will start from the lancet, or the application of ice; and he possesses some power to move the muscles. Nothing of all this happens in solar asphyxia. Effusion of blood upon the brain, extravasation into its ventricles or substance, as well as congestion, are much more gradual in their effects. The depression of large portions of the skull, and the presence of enormous clots of blood upon the brain, do not destroy all sensibility. In trephining the skull, while the patient seemed in a deep, snoring sleep, I have sometimes found it necessary to cause his hands to be held down, as he evidently felt pain from the operation.

The worst cases of apoplexy last from twelve to twenty-four hours, generally; solar asphyxia, as many minutes. Apoplexy affects one side, or half of the body, with palsy, and sometimes is attended with convulsion. After the apoplectic fit goes off, the palsy often remains; sometimes, permanently. Nothing of the kind occurs in solar asphyxia. In the former, the breathing is infrequent and snoring, the pulse is slow and hard; in the latter, the breathing is quick and rattling, the pulse rapid and gaseous. The former happens to the rich, the luxurious, the sedentary, the corpulent, the plethoric, those who have thick short necks; the latter happens to the poor, the laborer, the exposed, who undergo hardships. The former happens in cold weather and in hot, in the day time and at night; the latter, only in hot weather, and during the day. Solar asphyxia has no premonitory symptoms, except a sudden heat, and dryness of the skin; neither headache, scintillations, or any cerebral affection. Apoplexy has well known premonitory symptoms. The following passage is taken from the April No. (1841) of that treasury of medical science—Johnson's Med. Chir. Rev.—and is illustrative of this position. "Napoleon, who dreaded apoplexy, asked Corvisart, his first physician, for some information respecting this disease. Sire, replied Corvisart, apoplexy is always dangerous; but it is *always* preceded by certain symptoms; nature seldom strikes the blow without giving warning. A first attack, which is always slight, is a *summons without costs—sommation sans frais;*—a second, *summons with costs—sommation avec frais;*—but a third, *is an execution on the person—prise de corps.* Corvisart, himself, afforded a melancholy proof of the truth of his assertion."

Solar asphyxia is probably an universal lesion of the nervous system; but more particularly of that part which is necessary to the pulmonary circulation. The blood which suddenly accumulates in the lungs, ceases to be arterialized; it gorges not only the blood-vessels, but infiltrates the pulmonary substance, forming the most perfect example of hyperœmia, and even penetrates the coverings of the lungs,—*pleura pulmonalis*,—and is copiously effused into the cavity of the chest, as I have seen several times. In this contest, the lungs are, probably, throughout the attack, in a collapsed or passive state, permitting the blood not only to distend its proper vessels, but to permeate readily throughout the pulmonary texture, until the lung is, perhaps, one-fourth heavier than is usual. Whether the pulmonary congestion be the primary or secondary condition of insolation, I will not say; but I must remark, that of all morbid appearances, of a congestive character, this is the least equivocal, so far as I have examined. Here nothing is ambiguous; the congestion and hyperœmia, are truly wonderful.

Although physiology teaches us that man is endowed with the powers of maintaining the same heat of his body in all climates and situations, with few exceptions, still it is possible, under peculiar circumstances, that the body may become actually heated. A chemico-vital refrigeration, by means of perspiration or evaporation, is constantly going on—in health, especially—in hot climates. The "fire-kings" themselves, when in a heat of 500° or 600°, would roast and turn into cinders, were it not for this refrigerating process, in conjunction with a vital energy, which for a time neutralizes the accumulating power of caloric. The solar heat probably accumulates upon the surface of the body faster than nature can refrigerate through the lungs and the skin, by evaporation; inequilibrium presses upon the vital energy, which, being exhausted in the contest, as well as by excessive labor, is unable longer to neutralize the excess of temperature—often 40° more than that of the body. Vital chemistry is unequal to the task of preventing the conduction of heat into the body, and death is the consequence.—*N. Y. Med. Gaz., Oct.* 1841.

